# A Mediterranean Dietary Pattern Predicts Better Sleep Quality in US Women from the American Heart Association Go Red for Women Strategically Focused Research Network

**DOI:** 10.3390/nu12092830

**Published:** 2020-09-16

**Authors:** Faris M. Zuraikat, Nour Makarem, Marie-Pierre St-Onge, Huaqing Xi, Alekha Akkapeddi, Brooke Aggarwal

**Affiliations:** 1 Sleep Center of Excellence, Department of Medicine, Columbia University Irving Medical Center, New York, NY 10032, USA; fmz2105@cumc.columbia.edu (F.M.Z.); nm2968@cumc.columbia.edu (N.M.); ms2554@cumc.columbia.edu (M.-P.S.-O.); 2 Division of Cardiology, Department of Medicine, Columbia University Irving Medical Center, New York, NY 10032, USA; 3 Division of General Medicine, Department of Medicine, Columbia University Irving Medical Center, New York, NY 10032, USA; 4 Institute of Human Nutrition, Vagelos College of Physicians and Surgeons, Columbia University Irving Medical Center, New York, NY 10032, USA; aa4532@cumc.columbia.edu; 5 Department of Biostatistics, Columbia University Irving Medical Center, New York, NY 10032, USA; hx2263@cumc.columbia.edu

**Keywords:** Mediterranean diet, alternate Mediterranean diet pattern, sleep quality, sleep efficiency, sleep disturbances, women’s health

## Abstract

Consumption of a Mediterranean diet has been linked to better sleep health in older, European populations. However, whether this dietary pattern is predictive of sleep quality in US women, a group prone to poor sleep, is unknown. This prospective cohort study of 432 US women (20–76 y; 60% racial/ethnic minority) evaluated whether compliance with a Mediterranean diet at baseline predicted sleep quality at 1-y follow-up. Alternate Mediterranean (aMed) diet scores and habitual sleep quality were computed from the validated Block Brief Food Frequency Questionnaire and Pittsburgh Sleep Quality Index (PSQI), respectively. Linear regression models evaluated prospective associations of the aMed diet pattern and its components with measures of sleep quality, after adjustment for age, BMI, race/ethnicity, education, and health insurance status. Higher baseline aMed scores were associated with lower PSQI scores (β = −0.30 ± 0.10, *p* < 0.01), indicative of better sleep quality, higher sleep efficiency (β = 1.20 ± 0.35, *p* < 0.001), and fewer sleep disturbances (β = −0.30 ± 0.12, *p* = 0.01) at 1-y. Fruit and vegetable consumption also predicted lower PSQI scores, higher sleep efficiency and fewer sleep disturbances (all *p* < 0.05). Higher legume intake predicted better sleep efficiency (β = 1.36 ± 0.55, *p* = 0.01). These findings suggest that adherence to a Mediterranean diet pattern should be evaluated as a strategy to promote sleep quality in US women.

## 1. Introduction

Sleep and diet are key determinants of cardiovascular health (CVH) and are intricately related to one another [1,2]. Emerging data indicate that the association between sleep and diet is likely to be bidirectional [1,3]. On one hand, it is well-established that changes in sleep duration and quality can impact the types and amounts of food consumed [4]. Healthy sleep duration and markers of good sleep quality, including higher sleep efficiency and shorter sleep onset latency, have been linked with better diet quality, namely higher intakes of nuts, legumes, fruits, and unsaturated fat [5,6], as well as lower intakes of energy and added sugars [6,7]. Effects of short and poor sleep on food intake and diet quality have been demonstrated via mechanisms including greater sensitivity to food reward and changes in appetite hormones [8]. On the other hand, nascent data demonstrate that dietary patterns may influence sleep parameters; thus, a role for diet in sleep health has also been postulated [1,3,8]. However, far less research has investigated this aspect of the likely bidirectional sleep-diet relation, which is necessary for a more nuanced understanding of this association and to inform more effective interventions addressing these lifestyle factors for health promotion [1].

Results of small-scale trials demonstrate that supplementation with foods rich in melatonin, tryptophan, or other nutrients can impact parameters of sleep [9]. Recently, compelling data from the limited number of available epidemiological studies also demonstrate associations between dietary patterns and measures of sleep quantity and quality [1,10]. There is particular interest in the Mediterranean diet pattern for the promotion of sleep health [1]. Mediterranean diets are linked to favorable cardiovascular outcomes, and this is observed across multiple populations due to the adaptability of the diet [11]. For instance, consistent with the traditional Mediterranean diet, higher adherence to an alternate Mediterranean diet (aMed), adapted for US populations [12], is associated with lower risk of type 2 diabetes [13] and of cardiovascular diseases [14]. Most important to sleep health, these Mediterranean dietary patterns encourage consumption of nutrient-dense fruits and vegetables and foods high in plant-based protein and unsaturated fats [15], which are often rich in sleep-promoting nutrients and compounds [16].

Research in European adults showed that compliance with a Mediterranean diet was associated with better sleep quality and lower risk for insomnia [17,18,19], but these studies were cross-sectional, precluding determination of temporality. Findings from a prospective study conducted in older Spanish adults are consistent in demonstrating that greater adherence to a Mediterranean diet is related to more favorable sleep quality and lower risk of changes in sleep duration [20]. It is unclear if these findings would extend to US populations, given differential patterns of health behaviors and exposure to distinct psychosocial, environmental, and structural influences on health across cultures. To our knowledge, only one analysis, within the Multi-Ethnic Study of Atherosclerosis, evaluated the aMed diet in relation to sleep in US adults [21]. Although results showed that this diet pattern predicted lower odds of insomnia and short sleep, associations with measures of general sleep quality were not evaluated. Moreover, the populations for both of the aforementioned prospective studies were primarily older adults [20,21], which limits generalizability of findings to adults of other life stages.

The current study was designed to prospectively assess whether adherence to a Mediterranean diet modified for US populations (aMed) [12] predicts habitual sleep quality in US women, who generally report more sleep-related complaints than men [22]. Additionally, given previous cross-sectional associations of foods and nutrients encouraged in an aMed diet (e.g., fruits and vegetables, unsaturated fat) with parameters of sleep quality [6,17], we aimed to assess whether aMed diet components, or their key nutrients, were associated with future sleep quality. We hypothesized that greater concordance to the aMed diet at baseline would be associated with better overall sleep quality, highlighted by shorter sleep onset latency, higher sleep efficiency, and fewer sleep disturbances, at 1-y. We further hypothesized that higher baseline intakes of foods and nutrients encouraged in an aMed diet, and lower intakes of those that are discouraged, would predict better sleep quality. Given that poor sleep is related to poorer cardiovascular health (CVH) in women [2], identification of dietary patterns that promote sleep quality may help improve CVH in this population for which cardiovascular disease remains the leading cause of morbidity and mortality [23].

## 2. Materials and Methods

### 2.1. Participants and Procedures

Participants in this analysis were women enrolled in the population science study of the American Heart Association Go Red for Women Strategically Focused Research Network at Columbia University, a multi-study investigation of the role of sleep in CVH of women. The complete sample for the prospective cohort study was comprised of 506 non-pregnant women aged 20–76 y living in northern Manhattan and neighboring communities of New York City. All study visits were conducted at Columbia University Irving Medical Center (CUIMC)/New York-Presbyterian Hospital, with baseline visits occurring between July 2016 and January 2018, and 1-y follow-up between July 2017 and February 2019. Sociodemographic and lifestyle factors, including habitual patterns of diet and sleep, were evaluated using validated questionnaires administered in both English and Spanish by trained study staff. Anthropometrics were measured using standard procedures to reduce error [24]. Four hundred and fifty-six women returned for the 1-y follow-up visit (90% of total at baseline), and complete diet and sleep data, following removal of implausible intakes [25], were available from 432 (95%) of those women. All procedures were approved by the CUIMC Institutional Review Board (IRB; protocol number AAAQ8196) and conducted in accordance with the Declaration of Helsinki. Participants gave written informed consent prior to participation and were compensated following completion of the study.

### 2.2. Dietary Assessment

Habitual dietary intakes were assessed using the validated Block Brief Food Frequency Questionnaire (FFQ) [26,27]. Participants were asked to indicate the amount and frequency of consumption of ~70 common foods over the past year, and visual aids were provided to improve portion estimation. This tool has been validated against robust measures of dietary intake [27]. Data were sent to NutritionQuest (Berkeley, CA, USA) for nutrient analysis; diet variables related to a Mediterranean Diet pattern were obtained either directly from FFQ output or by summing two or more variables from the output. In addition, a total score for adherence to an alternate Mediterranean (aMed) diet was calculated based on the scale developed by Fung et al. for US populations [12]. We have previously described, in detail, computation of the aMed diet score using this dataset [28]. Briefly, median splits were performed for intakes of each of the nine components of the aMed diet: fruits, vegetables, legumes, nuts, dark breads (substitute for whole grains), fish, red and processed meat, proportion of monounsaturated fat (MUFA) to saturated fat (SFA), and alcohol. For all components other than red meat and alcohol, intakes above the median were given a score of 1. Intake of red meat below the median and alcohol between 5 and 15 g/d were each assigned a score of 1. Scores were summed for each participant, with a possible range of 0–9. Higher scores indicated greater adherence to an aMed diet pattern.

Daily intakes of food sources included in the aMed diet score were additional exposures of interest. Notably, when evaluated as an independent exposure, intakes of fruits and vegetables were summed. Consumption of key nutrients in aMed food sources, gathered from the FFQ output, were also evaluated as exposures of interest. These included intakes of MUFA, total unsaturated fat, SFA, plant protein, animal protein, and fiber. Diet variables were energy adjusted as follows: fruits and vegetables, legumes, nuts, dark breads, and red meat were calculated as servings/1000 kcal consumed; alcohol, unsaturated, and saturated fat were calculated as % of total kcal; and fish, plant protein, animal protein, and fiber were calculated as g/1000 kcal. Per recommendations for FFQ data [25], individuals with implausible intakes based on total reported energy (<400 kcal and >4000 kcal) were not included in analyses.

### 2.3. Sleep Assessment

Sleep quality was evaluated at baseline and 1-y follow-up using the validated Pittsburgh Sleep Quality Index (PSQI) [29]. This is the most widely used self-report measure of habitual sleep quality and has been shown to have internal consistency and reliability [30]. Overall sleep quality was determined by summing scores for the seven components comprising the PSQI; total scores range from 0–21 with higher scores indicating poorer overall sleep quality. Individual components of sleep quality, obtained from the PSQI, considered in these analyses included sleep onset latency (min), sleep efficiency (%), and sleep disturbances, as these are the key elements of sleep quality determined by the National Sleep Foundation [31]. Sleep onset latency was determined from the question “during the past month, how long (in minutes) has it usually taken you to fall asleep?”. Sleep efficiency was calculated by dividing the reported number of hours slept by the reported number of hours spent in bed (determined from habitual bed and wake times) and multiplying by 100; values over the maximum possible were treated as 100%. Sleep disturbance scores represented the composite frequency of experiencing trouble sleeping in response to nine different factors (waking up unexpectedly, nocturia, difficulty breathing, coughing/snoring, feeling too hot, feeling too cold, bad dreams, pain, or other); each factor received a score based on reported frequency and scores were summed across factors according to scoring guidelines [29].

### 2.4. Statistical Analysis

Descriptive characteristics of the sample are presented as mean ± standard deviation (SD) or as frequency and proportion of the total analytic sample. Paired samples t-tests were used to compare mean values of sleep quality measures between baseline and 1-y follow-up. Multivariable-adjusted linear regression models were used to evaluate whether exposure variables (aMed diet scores, intakes of foods and nutrients included in or related to an aMed diet), measured at baseline, were associated with outcome variables (total PSQI score, sleep onset latency, sleep efficiency, sleep disturbances), measured at 1-y follow-up. All exposure and outcome variables were assessed on the continuous scale. Models were adjusted for baseline age, body mass index (BMI), race/ethnicity, education level, and health insurance status (proxy for socioeconomic status). In additional models, the baseline sleep variable corresponding with the 1-y outcome was also included as a covariate. Results of regression analyses are presented as β ± standard error (SE). Sensitivity analyses for models on diet and sleep efficiency were conducted with individuals reporting sleep efficiency > 100% removed from the analytic sample in order to confirm whether results observed in the full sample persist. All analyses were conducted in SAS v9.4 (Cary, NC), and results are considered significant at *p* < 0.05.

## 3. Results

At baseline, average age and BMI of women included in the analytic samples were 37 ± 15 y and 25.9 ± 5.5 kg/m^2^, respectively (Table 1). Over half of the sample (60%) identified as a racial and/or ethnic minority. Baseline aMed scores ranged from 1.0 to 8.0, with a mean score of 4.3 ± 1.5. On average, women exceeded dietary recommendations for saturated fat intake (<10% kcal/d), did not meet recommendations for fiber (≥14 g/1000 kcal), and consumed more protein from animal than plant sources. Measures of sleep tended to improve from baseline to 1-y, with significant increases in overall sleep quality (5.5 ± 3.6 vs. 5.1 ± 3.3, *p* < 0.01) and reductions in sleep onset latency, in minutes, (24.3 ± 29.3 vs. 21.2 ± 21.0, *p* = 0.02) (Table 2).

Greater adherence to the aMed diet at baseline was associated with significantly lower PSQI scores, indicative of better sleep quality, at 1-y follow-up (β = −0.30 ± 0.10, *p* < 0.01; Table 3). Higher aMed scores also predicted lower sleep disturbance scores, indicative of fewer sleep disturbances (β = −0.30 ± 0.12, *p* = 0.01). In addition, each 1-point increase in baseline aMed score related to a 1.20 ± 0.35% higher sleep efficiency at 1-y (*p* < 0.001). In terms of food sources included in aMed scoring, legume consumption was associated with sleep efficiency at 1y (*p* = 0.01), whereby each 1 serving increase in legume consumption per 1000 kcal related to 1.36 ± 0.55% higher sleep efficiency (Table 3). In addition, higher intakes of fruits and vegetables at baseline predicted better overall sleep quality (β = −0.16 ± 0.07, *p* = 0.02), higher sleep efficiency (β = 0.56 ± 0.24, *p* = 0.02), and fewer sleep disturbances (β = −0.18 ± 0.08, *p* = 0.03). Results were similar in unadjusted models (Supplementary Table S1) as well as in models further adjusted for baseline sleep characteristics (Table 3). When evaluating baseline intakes of fruits and vegetables as separate predictors, results of fully-adjusted models showed that higher fruit intake predicted fewer sleep disturbances (β = −0.56 ± 0.23, *p* = 0.02), while higher vegetable intake predicted lower total PSQI scores (β = −0.22 ± 0.06, *p* < 0.001) and higher sleep efficiency (β = 0.66 ± 0.25, *p* < 0.01) at 1-y. Results of the sensitivity analysis were similar to the full sample, but associations of fruit and vegetable intake with sleep efficiency were slightly attenuated (*p* = 0.06).

Intakes of major nutrients from food sources included in the aMed diet score also predicted measures of sleep quality (Table 4). For instance, a 1-point increase in MUFA to SFA ratio related to a 3.11 ± 1.43% higher sleep efficiency at 1 y (*p* = 0.03). Correspondingly, higher total intakes of unsaturated fat at baseline were associated with lower total PSQI scores (β = −0.07 ± 0.03, *p* = 0.02) as well as shorter sleep onset latency (β = −0.48 ± 0.19, *p* = 0.01) after 1 y. Consumption of plant protein and fiber at baseline predicted sleep efficiency at 1-y follow-up (both *p* < 0.01); 1 g/1000 kcal increases in plant protein and fiber were associated with a 0.99 ± 0.31% and 0.33 ± 0.12% higher sleep efficiency, respectively. Notably, further adjustment for baseline sleep characteristics resulted in additional significant associations of diet with sleep; higher baseline fiber and plant protein intakes were associated with lower 1-y total PSQI scores, indicative of better sleep quality, and fewer sleep disturbances (Table 4). In the sensitivity analysis, results were similar to those in the full sample, although the association of MUFA to SFA ratio with 1-y sleep efficiency was attenuated in the model not adjusted for baseline sleep efficiency (*p* = 0.07) and strengthened in the model adjusted for baseline sleep efficiency (*p* = 0.02).

## 4. Discussion

Herein, we provide some of the earliest evidence that greater concordance with an aMed diet pattern predicts better sleep quality in a diverse sample of US women. This study also represents, to our knowledge, the first prospective evaluation of the association of the components of an aMed diet with measures of sleep quality, demonstrating that higher intakes of nutrient-dense fruits and vegetables, legumes, unsaturated fat, and plant-based proteins are related to better future sleep quality. Taken together, these results provide novel insight into the role of a Mediterranean dietary pattern in parameters of sleep health that extend beyond sleep duration or sleep disorders [20,21] and identify specific dietary components that may contribute most to good sleep. The observed aMed diet-sleep quality link elucidates sleep as a possible underlying mechanism in the well-documented association between the aMed diet and lower risk of cardiovascular disease [14].

Adherence to a Mediterranean diet is increasingly associated with health benefits [28,32], and results of the current study indicate that this also includes better habitual sleep quality. A prospective relation between the Mediterranean diet and sleep health was first established in Spanish men and women over 60 y old from the Seniors-Study on Nutrition and Cardiovascular Risk (ENRICA) cohort [20]; results showed that higher scores on the Mediterranean Diet Adherence Screener (MEDAS) related to lower risk of reporting multiple indicators of poor sleep. Findings of the current investigation provide an important extension to that study by corroborating this relation in US individuals, whose habitual dietary patterns are distinct from Europeans, as reflected by different measures of the Mediterranean diet tailored for use in the respective populations (MEDAS vs. aMed). Our findings are also consistent with those of the only other study conducted in a US population, which showed that greater compliance with an aMed diet predicted lower risk for insomnia in the Multi-Ethnic Study of Atherosclerosis [21]. The current study, however, provides unique insight beyond that of any previous investigation by identifying specific components of sleep quality to which this Mediterranean diet-sleep relation extends, including higher sleep efficiency and fewer sleep disturbances. Importantly, this study is the earliest report of these associations in a diverse cohort of US women spanning a broad range of ages, which has public health relevance given that sleep complaints are widely prevalent across all stages of adulthood in women [33].

A key element of the aMed diet is high intakes of plant-based foods [15], including fruits and vegetables and legumes, which have been hypothesized to promote sleep quality [16]. We show here that greater consumption of these foods is predictive of markers of better sleep quality, including higher sleep efficiency. These findings support associations of fruit and vegetable intake with sleep quality in cross-sectional studies [17,34], and suggest a potential for increased fruit and vegetable intake to improve sleep quality, although experimental research is needed to confirm this. The high nutrient density of fruits, vegetables, and legumes may underlie their associations with sleep quality. These foods are rich in fiber [35], which we find relates to better overall sleep quality, higher sleep efficiency, and fewer sleep disturbances after 1 y in this cohort. This loosely corresponds with observation of an increase in time spent in deep sleep following higher fiber intake over the previous day [36]. Associations of fruits and vegetables and legumes with future sleep quality in this study may also help to explain previous findings linking lower dietary glycemic index with reduced risk of insomnia symptoms [10], since fiber-rich foods such as these can lower overall dietary glycemic index [37]. A potential sleep quality-promoting role of fruits and vegetables, indicated in our findings, adds to the various health benefits of this food group, including weight management [38] and reduced cardiovascular disease risk [39]. Furthermore, data from this study identify fruits and vegetables as drivers, at least in part, of the observed relation between an aMed diet and sleep quality, further emphasizing their role as a key component of the diet for health promotion.

For components other than fruits and vegetables, results of this study indicate that associations of an aMed diet with sleep may be driven to a greater extent by total intakes of nutrients across sources, rather than by individual foods or food groups. For example, baseline intakes of red and processed meats were not related to overall sleep quality nor its components in this cohort. This contrasts with a previous study in older Spanish adults from the Seniors-ENRICA cohort [40], which found that higher total meat intake was associated with increased odds of poor sleep. Differences may be explained by our focus on red meat, rather than total meat intake, or by a narrower range of meat consumption in the current cohort, driven by lower red meat intakes. It could also be that associations with sleep differ by meat source or type of processing (e.g., processed meats vs. red meats); this will need to be elucidated in future research. While we did not observe inverse associations of red meat intake with parameters of sleep quality, higher intakes of protein from plant sources did predict higher sleep efficiency in these women. Thus, data still suggest a potential benefit of increasing the proportion of protein consumed from plant compared to animal sources, as is encouraged in the Mediterranean diet [15]. In addition, based on previous findings of a beneficial influence of fish intake on sleep onset latency [41], we had also hypothesized that higher fish intake would predict better sleep quality. This hypothesis was not supported; however, we did find that a higher MUFA to SFA ratio and greater total intakes of unsaturated fat, of which fatty fish are a rich source, are linked to better future sleep quality, extending a cross-sectional association of unsaturated fat with overall sleep quality [6]. Together, these findings help to explain how a Mediterranean diet can improve sleep quality, but also suggest that, for promotion of sleep, recommendations for this diet should extend to a broader range of foods rich in unsaturated fat and plant-based protein. It would also be of interest to investigate other diet indices that promote intakes of similar nutrient sources, such as the Healthy Eating Index, to determine if there is a diet pattern most predictive of healthy sleep in US populations.

A number of possible mechanisms could underlie the associations of a Mediterranean diet with sleep quality metrics. One contributing factor could be increased consumption of sleep-promoting compounds, including tryptophan and melatonin, via the Mediterranean diet [16]. Many plant-based foods, including fruits and vegetables, are good sources of melatonin, and clinical trials demonstrate that consumption of these foods can improve parameters related to sleep quality, such as sleep disturbances [3]. Green leafy vegetables are also a rich source of nitrates that convert to nitric oxide when consumed; the Mediterranean diet contains significantly higher amounts of nitrate than the typical Western diet [42]. Reduced nitric oxide bioavailability may contribute to endothelial dysfunction [43], which has been linked to poor sleep in women [44]. Similarly, legumes are often rich in the amino acid tryptophan, which is a precursor for melatonin. Another potential pathway by which the diet can improve sleep quality is the microbiome. As we have discussed in a previous review [1], food sources encouraged by a Mediterranean diet pattern, including fruits and vegetables, are often rich sources of fiber and other nutrients that can have beneficial effects on the microbial composition of the gut, such as a lower ratio of Firmicutes to Bacteroidetes [1,45]. In turn, markers of healthy composition of gut flora have been linked with better sleep [46]. However, physiological mechanisms underlying the aMed diet-sleep quality association require further investigation, possibly using clinical investigations that evaluate the effect of an aMed diet intervention on sleep quality and corresponding changes in microbial composition or circulating melatonin.

This study in a diverse sample of women enhances our foundational understanding of the contribution of a widely recommended healthy diet pattern to good sleep quality, which has been linked in this cohort to more favorable CVH [2]. One strength is the prospective design, which allowed for establishment of temporality. Although the sample size was moderate, the study population included women across all stages of adulthood, thereby enhancing generalizability of findings. An essential next step will be to evaluate associations of the Mediterranean diet as a predictor of sleep quality in a larger sample with sufficient power for pertinent subgroup analyses, so that potential moderating roles of race/ethnicity as well as menopausal and caregiving status can be evaluated. This is important given evidence that racial/ethnic minorities, post-menopausal women, and individuals with caregiving responsibilities are at increased risk for poor sleep [33,47,48]. Similarly, there is a need to evaluate potential sex differences in the relation between a Mediterranean diet and sleep. Although we demonstrate strong associations in this cohort of women, null results were reported in a previous study of older men [49]. Thus, sex-specific relations between diet and sleep, and potential underlying mechanisms, warrant investigation in future cohort studies that include both men and women. The observed associations in this study also warrant confirmation using objective measures of diet and sleep quality, enabling more accurate estimation of these factors. Use of objective measures of sleep, such as actigraphy or polysomnography, would also allow for evaluation of sleep phenotypes that could not be assessed using the PSQI. Despite limitations of self-reported measures, it is notable that both diet and sleep assessment tools used in this study are validated and widely used [27,29,30]. Objective measures of food intake would not have been feasible given the sample size, and FFQ are often used for determination of aMed diet adherence [12]. In addition, we have previously shown that values on the PSQI can correlate with objective measures of sleep [50].

Sleep quality tends to decline with age in women [33], putting them at risk for adverse health outcomes including obesity and cardiovascular disease [51,52]. Data from this study identify increased adherence to an aMed diet pattern and intake of its major components, namely fruits, vegetables, and plant-based foods rich in protein and unsaturated fats, as a possible lifestyle intervention to promote sleep quality in women. Improving sleep health in response to adopting a Mediterranean diet could augment the direct benefits of this diet on CVH and help to sustain healthful changes to the diet, due to the bidirectional relation of diet and sleep, underscored by better appetite regulation during good sleep [53]. Thus, our findings suggest that adherence to a Mediterranean diet pattern may be warranted for public messaging to promote sleep health in women.

## Figures and Tables

**Table 1:** Descriptive characteristics of the analytic sample at baseline (*n* = 432).

Characteristic	Mean ± SD/*n* (%)
Demographic and physical	
Age (years)	37 ± 15
Race	
White	247 (57)
Black/African American	84 (19)
Asian	80 (19)
Other	21 (5)
Race/Ethnicity	
White/Non-Hispanic	172 (40)
Minority/Hispanic	260 (60)
Health Insurance	
Private/Medicare	276 (64)
Do not have/Unknown/Medicaid	156 (36)
Education	
>College degree	139 (32)
≤College degree	293 (67)
Body Mass Index (BMI) (kg/m^2^)	25.9 ± 5.5
<25 kg/m^2^	232 (54)
≥25 kg/m^2^	200 (46)
**Dietary Intakes**	
Alternate Mediterranean Diet (aMed) Score	4.3 ± 1.5
Fruits and vegetables ^a^	3.8 ± 2.3
Legumes ^a^	0.9 ± 1.0
Nuts ^a^	0.7 ± 0.8
Dark breads ^a^	0.3 ± 0.4
Red/processed meat ^a^	1.2 ± 1.3
Fish ^b^	12.6 ± 15.0
MUFA to SFA ratio	1.3 ± 0.4
Alcohol ^c^	4.4 ± 5.6
Unsaturated fat ^c^	23.5 ± 5.3
Saturated fat ^c^	12.7 ± 3.0
Plant protein ^b^	5.9 ± 1.7
Animal protein ^b^	10.0 ± 3.8
Fiber ^b^	11.5 ± 4.5

^a^ Servings consumed per 1000 kcal. ^b^ Grams consumed per 1000 kcal. ^c^ Percent of total kcal. SD: Standard Deviation.

**Table 2:** Measures of sleep of the analytic sample at baseline and 1-y follow-up.

Sleep Characteristic	Baseline	1-y Follow-Up	*p*-Value
**Total PSQI score ^a^**	5.5 ± 3.6	5.1 ± 3.3	<0.01
**Sleep onset latency (min) ^b^**	24.3 ± 29.3	21.2 ± 21.0	0.02
**Sleep efficiency (%) ^b^**	88.3 ± 11.4	87.5 ± 11.3	0.24
**Sleep disturbance score ^b^**	6.1 ± 4.4	5.8 ± 4.2	0.05

^a^ Possible range of scores is 0–21, with higher scores indicating poorer sleep quality. ^b^ Component of the Pittsburgh Sleep Quality Index (PSQI) calculated using scoring instructions from the original manuscript.

**Table 3:** Prospective analysis of associations of baseline adherence to an alternate Mediterranean (aMed) diet and intake of food sources included in the aMed score with measures of sleep quality after 1-y ^a^.

Predictor	Outcome	β (SE) ^b^	*p*-Value	β (SE) ^c^	*p*-Value
**aMed diet score**	PSQI total score	−0.30 (0.10)	<0.01	−0.31 (0.08)	<0.0001
	Sleep onset latency	−0.61 (0.65)	0.35	−0.71 (0.59)	0.23
	Sleep efficiency	1.20 (0.35)	<0.001	1.21 (0.33)	<0.001
	Sleep disturbances	−0.30 (0.12)	0.01	−0.35 (0.10)	<0.001
**Fruits and vegetables**	PSQI total score	−0.16 (0.07)	0.02	−0.19 (0.05)	<0.001
	Sleep onset latency	−0.41 (0.44)	0.36	−0.31 (0.40)	0.44
	Sleep efficiency	0.56 (0.24)	0.02	0.52 (0.22)	0.02
	Sleep disturbances	−0.18 (0.08)	0.03	−0.15 (0.07)	0.02
**Legumes**	PSQI total score	−0.10 (0.16)	0.55	−0.24 (0.13)	0.06
	Sleep onset latency	−1.13 (1.03)	0.27	−1.21 (0.94)	0.20
	Sleep efficiency	1.36 (0.55)	0.01	1.46 (0.52)	<0.01
	Sleep disturbances	0.17 (0.19)	0.39	−0.08 (0.16)	0.62
**Nuts**	PSQI total score	0.01 (0.21)	0.96	0.02 (0.17)	0.92
	Sleep onset latency	0.09 (1.35)	0.95	0.25 (1.23)	0.84
	Sleep efficiency	−0.47 (0.72)	0.51	−0.36 (0.68)	0.60
	Sleep disturbances	−0.26 (0.25)	0.31	−0.09 (0.20)	0.65
**Dark breads**	PSQI total score	−0.68 (0.39)	0.08	−0.55 (0.30)	0.07
	Sleep onset latency	−0.94 (2.48)	0.71	−1.09 (2.26)	0.63
	Sleep efficiency	2.07 (1.33)	0.12	1.96 (1.26)	0.12
	Sleep disturbances	−0.43 (0.47)	0.36	−0.67 (0.38)	0.08
**Fish**	PSQI total score	0.00 (0.01)	0.99	−0.004 (0.01)	0.67
	Sleep onset latency	0.02 (0.07)	0.74	−0.02 (0.06)	0.73
	Sleep efficiency	−0.01 (0.04)	0.73	−0.01 (0.03)	0.76
	Sleep disturbances	−0.01 (0.01)	0.32	−0.01 (0.01)	0.36
**Red/processed meat**	PSQI total score	−0.02 (0.12)	0.89	0.07 (0.10)	0.49
	Sleep onset latency	−0.00 (0.80)	0.99	0.22 (0.73)	0.76
	Sleep efficiency	−0.06 (0.43)	0.89	−0.28 (0.41)	0.49
	Sleep disturbances	0.04 (0.15)	0.81	0.04 (0.12)	0.74

^a^ Results of linear models represent the change in total Pittsburgh Sleep Quality Index (PSQI) score (points), sleep onset latency (m), sleep efficiency (%), or sleep disturbances per 1 point increase in aMed score or 1 serving increase in intake of fruits and vegetables, legumes, nuts, dark breads, fish (g), and red meat (per 1000 kcal). ^b^ Multivariable linear regression models adjusted for age, BMI, race/ethnicity, education, and health insurance status. ^c^ Multivariable linear regression models adjusted for corresponding sleep variable at baseline, age, BMI, race/ethnicity, education, and health insurance status. SE: Standard Error.

**Table 4:** Prospective analysis of associations of baseline intakes of major nutrients in food sources in the alternate Mediterranean (aMed) diet with parameters of sleep quality at 1-y follow-up ^a^.

Predictor	Outcome	β (SE) ^b^	*p*-Value	β (SE) ^c^	*p*-Value
**Monounsaturated fat (MUFA) to saturated fat (SFA) ratio**	PSQI total score	−0.84 (0.41)	<0.05	−0.38 (0.33)	0.25
	Sleep onset latency	−2.26 (2.69)	0.40	−1.10 (2.45)	0.65
	Sleep efficiency	3.11 (1.43)	0.03	2.40 (1.36)	0.08
	Sleep disturbances	−0.95 (0.50)	0.06	−0.56 (0.40)	0.17
**Unsaturated fat**	PSQI total score	−0.07 (0.03)	0.02	−0.02 (0.02)	0.35
	Sleep onset latency	−0.48 (0.19)	0.01	−0.43 (0.17)	0.01
	Sleep efficiency	0.13 (0.10)	0.22	0.09 (0.10)	0.38
	Sleep disturbances	−0.05 (0.04)	0.19	−0.01 (0.03)	0.75
**Saturated fat**	PSQI total score	−0.004 (0.05)	0.93	0.04 (0.04)	0.35
	Sleep onset latency	−0.47 (0.33)	0.16	−0.46 (0.30)	0.14
	Sleep efficiency	−0.09 (0.18)	0.63	−0.07(0.17)	0.70
	Sleep disturbances	0.06 (0.06)	0.33	0.10 (0.05)	0.05
**Plant protein**	PSQI total score	−0.14 (0.09)	0.14	−0.20 (0.07)	<0.01
	Sleep onset latency	−0.06 (0.59)	0.92	−0.15 (0.54)	0.78
	Sleep efficiency	0.99 (0.31)	<0.01	0.93 (0.30)	<0.01
	Sleep disturbances	−0.13 (0.11)	0.26	−0.18 (0.09)	<0.05
**Animal protein**	PSQI total score	−0.02 (0.04)	0.66	−0.003 (0.03)	0.92
	Sleep onset latency	−0.16 (0.26)	0.54	−0.09 (0.24)	0.71
	Sleep efficiency	0.02 (0.14)	0.87	−0.01 (0.13)	0.92
	Sleep disturbances	−0.05 (0.05)	0.28	−0.03 (0.04)	0.47
**Fiber**	PSQI total score	−0.06 (0.04)	0.08	−0.09 (0.03)	<0.01
	Sleep onset latency	−0.20 (0.22)	0.38	−0.18 (0.20)	0.39
	Sleep efficiency	0.33 (0.12)	<0.01	0.34 (0.11)	<0.01
	Sleep disturbances	−0.07 (0.04)	0.09	−0.09 (0.03)	<0.01
**Alcohol**	PSQI total score	0.05 (0.03)	0.06	0.04 (0.02)	0.05
	Sleep onset latency	0.25 (0.18)	0.17	0.33 (0.17)	<0.05
	Sleep efficiency	−0.16 (0.10)	0.11	−0.15 (0.09)	0.10
	Sleep disturbances	0.05 (0.03)	0.12	0.03 (0.03)	0.37

^a^ Results of linear models represent the change in total Pittsburgh Sleep Quality Index (PSQI) score (points), sleep onset latency (m), sleep efficiency (%), or sleep disturbances per 1 point increase in MUFA to SFA ration, 1% increase in kcal from unsaturated and saturated fat, or 1 g increase in animal protein, plant protein, and fiber intakes (per 1000 kcal). ^b^ Multivariable linear regression models adjusted for age, body mass index (BMI), race/ethnicity, education, and health insurance status. ^c^ Multivariable linear regression models adjusted for corresponding sleep variable at baseline, age, BMI, race/ethnicity, education, and health insurance status. SE: Standard Error.

## Supplementary Materials

The following are available online at https://www.mdpi.com/2072-6643/12/9/2830/s1, Table S1: Prospective associations of an aMed dietary pattern with measures of sleep quality after 1-y (unadjusted models).
