# Supplementary material for: A Mediterranean Dietary Pattern Predicts Better Sleep Quality in US Women from the American Heart Association Go Red for Women Strategically Focused Research Network
Source: Nutrients. 2020 Sep 16;12(9):2830. doi: 10.3390/nu12092830 (PMC7551612; doi:10.3390/nu12092830)
Supplement: Supplementary file 1 [file nutrients-12-02830-s001.pdf]

**Supplementary Table 1.** Prospective associations of an aMed dietary pattern with measures of sleep quality after 1-y (unadjusted models) [a]

| Predictor | Outcome | β (SE) | P-value |
|---|---|---|---|
| **Overall diet score and food sources included in the diet score** | | | |
| **aMed diet score** | PSQI total score | -0.25 (0.10) | **0.02*** |
| | Sleep onset latency | -0.45 (0.66) | 0.50 |
| | Sleep efficiency | 1.06 (0.35) | **<0.01*** |
| | Sleep disturbances | -0.22 (0.13) | 0.09 |
| **Fruits and vegetables** | PSQI total score | -0.16 (0.07) | **0.02*** |
| | Sleep onset latency | -0.47 (0.44) | 0.31 |
| | Sleep efficiency | 0.58 (0.24) | **0.01*** |
| | Sleep disturbances | -0.17 (0.09) | 0.05 |
| **Legumes** | PSQI total score | -0.02 (0.16) | 0.92 |
| | Sleep onset latency | -0.86 (1.03) | 0.40 |
| | Sleep efficiency | 1.09 (0.55) | **<0.05*** |
| | Sleep disturbances | 0.27 (0.21) | 0.19 |
| **Nuts** | PSQI total score | 0.09 (0.22) | 0.66 |
| | Sleep onset latency | 0.33 (1.36) | 0.81 |
| | Sleep efficiency | -0.73 (0.73) | 0.31 |
| | Sleep disturbances | -0.14 (0.27) | 0.61 |
| **Dark breads** | PSQI total score | -0.58 (0.39) | 0.14 |
| | Sleep onset latency | -0.23 (2.48) | 0.93 |
| | Sleep efficiency | 2.00 (1.33) | 0.13 |
| | Sleep disturbances | -0.13 (0.50) | 0.79 |
| **Fish** | PSQI total score | 0.01 (0.01) | 0.57 |
| | Sleep onset latency | 0.03 (0.07) | 0.68 |
| | Sleep efficiency | -0.03 (0.04) | 0.44 |
| | Sleep disturbances | -0.00 (0.01) | 0.90 |
| **Red meat** | PSQI total score | -0.03 (0.13) | 0.79 |
| | Sleep onset latency | -0.19 (0.80) | 0.81 |
| | Sleep efficiency | -0.08 (0.43) | 0.85 |
| | Sleep disturbances | 0.00 (0.16) | 0.99 |
| **Major nutrients in food sources included in the aMed diet score** | | | |
| **MUFA to SFA ratio** | PSQI total score | -0.84 (0.42) | **<0.05*** |
| | Sleep onset latency | -2.86 (2.68) | 0.29 |
| | Sleep efficiency | 2.80 (1.44) | 0.05 |
| | Sleep disturbances | -1.01 (0.53) | 0.06 |
| **Unsaturated fat** | PSQI total score | -0.07 (0.03) | **0.03*** |
| | Sleep onset latency | -0.49 (0.19) | **<0.01*** |

| | | | |
|---|---|---|---|
| | Sleep efficiency | 0.12 (0.10) | 0.25 |
| | Sleep disturbances | -0.05 (0.04) | 0.23 |
| **Saturated fat** | PSQI total score | -0.02 (0.05) | 0.77 |
| | Sleep onset latency | -0.45 (0.33) | 0.17 |
| | Sleep efficiency | -0.02 (0.18) | 0.89 |
| | Sleep disturbances | 0.06 (0.07) | 0.41 |
| **Plant protein** | PSQI total score | -0.17 (0.09) | 0.08 |
| | Sleep onset latency | -0.13 (0.60) | 0.82 |
| | Sleep efficiency | 1.07 (0.32) | **<0.001*** |
| | Sleep disturbances | -0.16 (0.12) | 0.17 |
| **Animal protein** | PSQI total score | -0.01 (0.04) | 0.80 |
| | Sleep onset latency | -0.16 (0.26) | 0.55 |
| | Sleep efficiency | 0.00 (0.14) | 0.99 |
| | Sleep disturbances | -0.03 (0.05) | 0.60 |
| **Fiber** | PSQI total score | -0.06 (0.04) | 0.07 |
| | Sleep onset latency | -0.20 (0.23) | 0.37 |
| | Sleep efficiency | 0.34 (0.12) | **<0.01*** |
| | Sleep disturbances | -0.07 (0.05) | 0.11 |
| **Alcohol** | PSQI total score | 0.02 (0.03) | 0.42 |
| | Sleep onset latency | 0.15 (0.18) | 0.42 |
| | Sleep efficiency | -0.08 (0.10) | 0.43 |
| | Sleep disturbances | 0.01 (0.04) | 0.72 |

[a] Univariate linear regression models with no adjustment for confounding variables
